# Common variants of *HTR3* genes are associated with obsessive-compulsive disorder and its phenotypic expression

**DOI:** 10.1038/srep32564

**Published:** 2016-09-12

**Authors:** Hae Won Kim, Jee In Kang, Sang-Hyuk Lee, Suk Kyoon An, Sung Yun Sohn, Eun Hee Hwang, Su Young Lee, Se Joo Kim

**Affiliations:** 1 Department of Psychiatry, Yonsei University College of Medicine, Seoul, Republic of Korea; 2 Institute of Behavioral Science in Medicine, Yonsei University College of Medicine, Seoul, Republic of Korea; 3 Department of Psychiatry, CHA Bundang Medical Center, CHA University, Seongnam, Republic of Korea; 4 Department of Psychiatry, Cheil General Hospital & Women’s Healthcare Center, Dankook University College of Medicine, Seoul, Republic of Korea

## Abstract

Evidence from literature supports the existence of associations between serotonin-related genetic variants and obsessive-compulsive disorder (OCD), but few studies have explored the involvement of serotonin receptor type 3 genes (*HTR3*) in OCD. To identify whether *HTR3* variability affects an individual’s susceptibility to OCD, we examined 10 *HTR3* variants in 596 individuals with OCD and 599 controls. A significant difference existed in the genotypic distribution of the *HTR3B* variant rs1176744 between individuals with OCD and controls (odds ratio [OR] = 0.74, 95% confidence interval [CI] = 0.60–0.91, *P* = 0.0043). A protective haplotype in *HTR3B* was also associated with OCD (OR = 0.77, CI = 0.63–0.95, permutated *P* = 0.0179). Analyses of OCD sub-phenotypes demonstrated significant associations between rs3758987 and early onset OCD in male subjects (OR = 0.49, CI = 0.31–0.79, *P* = 0.0031) and among rs6766410, rs6443930, and the cleaning dimension in female subjects (OR = 0.36, CI = 0.18–0.69, *P* = 0.0016 and OR = 0.47, CI = 0.29–0.79, *P* = 0.0030, respectively). Additionally, rs6766410 was related to contamination-based disgust in OCD (*P* = 0.0044). These results support that common *HTR3* variants are involved in OCD and some of its clinical phenotypes.

Obsessive-compulsive disorder (OCD) is often familial, and findings from twin and family studies have shown that obsessive-compulsive symptoms are substantially heritable, with a complex pattern of inheritance^1^,^2^. Given the evidence in support of a genetic aetiology for OCD, numerous candidate gene association studies have been conducted with genetic variants relevant to the pathways for serotonin, dopamine, and glutamate^3^. Polymorphisms related to serotonergic neurotransmission have been the most frequently examined owing to the clinical benefits of selective serotonin reuptake inhibitors (SSRIs) in the treatment of OCD. Indeed, a recent meta-analysis suggested that variations in two serotonin-related genes, *5-HTTLPR* and *HTR2A*, are associated with OCD^3^, while clearer evidence regarding the effects of other serotonin-related gene variants remains to be found.

Among the several potentially susceptible genes, various studies support the involvement of serotonin receptor type 3 (5-HT_3_) genes (*HTR3*) in the development of OCD. The 5-HT_3_ receptor is a Cys-loop ligand-gated cation channel that, when activated, mediates rapid depolarizing responses in neurons^4^. Along with the well-established role of these receptors in nausea and emesis, a recent study has suggested that activation of 5-HT_3_ receptors in the posterior insular cortex may enhance conditioned disgust behaviours in rats^5^. Considering that heightened disgust sensitivity appears to contribute to contamination concerns and washing rituals in individuals with OCD^6^ and in non-clinical samples^7^, 5-HT_3_ receptors seem to play a pathophysiological role in at least some types of OCD. In addition, these receptors reportedly affect cognitive and emotional functions, which may be explained by their influence on the release of various neurotransmitters^8^,^9^,^10^ in brain regions such as the hippocampus, amygdala, striatum, and nucleus accumbens. In line with this preclinical evidence, clinical trials of 5-HT_3_ antagonists have demonstrated efficacy in reducing symptoms of OCD^11^,^12^,^13^,^14^. Recently, a randomized, double-blind, placebo-controlled study revealed that 5-HT_3_ antagonists may offer additional clinical benefits when given in combination with SSRIs^15^.

To date, five distinct *HTR3* genes have been cloned for humans: *HTR3A* and *B* are located on chromosome 11q23.1–2^16^ while *HTR3C*, *D*, and *E* are located on chromosome 3q27.1^17^. A large genome-wide linkage study for OCD provided evidence that OCD is linked to markers on chromosome 3q27–28, although the findings did not reach the accepted level of statistical significance^18^. Considering that the markers are 2.5 Mb downstream of *HTR3C*–*E*, these genes may be positional candidates in OCD. Additionally, several authors have indicated that the single nucleotide polymorphism (SNP) rs1062613 in *HTR3A* is associated with the personality trait of harm avoidance^19^ and the modulation of amygdala activation^20^ in healthy subjects, both of which are suggested to have particular relevance for OCD^21^. *HTR3* genes may therefore be plausible candidates with regard to their involvement in OCD. To the best of our knowledge, however, only two association studies have investigated the involvement of *HTR3* in OCD. In a study with 75 trio samples, no significant association was found between the *HTR3A* variant rs1062613 and early onset OCD^22^,^23^. The other study utilized case-control samples and demonstrated that the *HTR3E* variant rs7627615 was related to the washing dimension and visual organization scores in OCD^24^.

Given this paucity of data and the promising clinical outcomes that are being achieved in subjects with OCD following the use of 5-HT_3_ antagonists, we aimed to perform a case-control association study with common *HTR3* variants in a larger sample of adult OCD probands and controls. Clinical characteristics such as the onset age and symptom dimensions were included in the analyses in terms of their relationship to the genetic variants, as these phenotypes have been proposed as a means of determining subgroups that are more genetically valid^25^. Furthermore, we sought to establish whether variability in *HTR3* contributes to disgust sensitivity, a psychological trait closely associated with OCD^6^. We hypothesized that variations within *HTR3* may confer genetic vulnerability to OCD and its associated clinical characteristics and psychological traits.

## Results

### Genotyping quality control

The threshold for the genotyping call rate was set at 95% for each SNP, with an average call rate of 99.2%. None of the SNPs in controls, individuals with OCD, or the entire sample significantly deviated from the Hardy-Weinberg equilibrium at a Bonferroni-corrected significance level of α = 0.005. The minor allele frequencies were >0.05 for all SNPs. Table 1 provides a detailed description of each SNP.

### Subjects

As shown in Table 2, no significant differences were found regarding the sex distribution or years of education between the two groups, but individuals with OCD were significantly older than were controls. Of the individuals with OCD, 103 (17.3%) were drug-naïve at enrolment. As for the disgust sensitivity trait, both groups demonstrated similar scores for core disgust, whereas individuals with OCD scored significantly higher on animal reminder disgust and contamination-based disgust.

### Single SNP association analysis

Regarding the genotype distributions, four SNPs were nominally significantly different between individuals with OCD and controls: rs1062613, rs3758987, rs1176744, and rs3782025. However, only rs1176744 remained significantly different after Bonferroni correction under an additive model (Table 3).

The analyses based on the subjects’ clinical characteristics, including their onset age and symptom dimensions, yielded no significant results for the entire OCD sample. However, significant associations were observed in a further analysis stratified by sex. Considering the onset age, the genotype distribution of rs3758987 differed significantly between male subjects with early onset OCD and male subjects with late onset OCD under a dominant model (Table 4). For the analysis of symptom dimensions, two SNPs, rs6766410 and rs6443930, were significantly associated with the cleaning dimension in female subjects under an overdominant model and additive model, respectively (Table 5). To determine whether these associations were independent, we performed a conditional analysis between these two SNPs. The results of conditional analysis remained significant, which were consistent with the low linkage disequilibrium (LD) between these SNPs (D′ = 0.05 and *r*^2^ = 0). The results of this conditional analysis are provided in Supplementary Table S1.

Concerning the relationship between disgust sensitivity and *HTR3* variants in OCD, we found a nominally significant effect of rs6766410 on the combined disgust scale-revised (DS-R) scores under an overdominant model (*F* [3,252] = 3.472, *P* = 0.0167, Wilk’s λ = 0.960, partial η^2^ = 0.040). A follow-up univariate analysis of variance revealed that the contamination-based disgust scores were significantly lower in subjects with the AC genotype than they were in subjects with the AA/CC genotypes (8.42 ± 3.98 and 9.85 ± 3.98, respectively; *F* [1,254] = 8.251, *P* = 0.0044, partial η^2^ = 0.031). No significant associations were found between the other *HTR3* variants and disgust sensitivity scores in OCD.

### Haplotype association analysis

We identified four LD blocks, three of which contained two markers from each gene and one of which contained three markers from *HTR3C* and *HTR3E* (Supplementary Figure S1). For the haplotypes estimated in *HTR3B*, a significant difference was observed for the distribution of haplotypes between individuals with OCD and controls. As shown in Table 6, a specific haplotype C-C was significantly associated with a lower risk of being affected by OCD. For the haplotypes in the other *HTR3* genes, no evidence of a relationship with OCD was found.

## Discussion

Here, we explored whether *HTR3* genetic variants confer risk for OCD and/or for certain clinical characteristics of the disorder. Our results support the involvement of *HTR3* in OCD, both in the onset age and in the manifestation of specific symptom dimensions.

We found a global relationship between the *HTR3B* variant rs1176744 and OCD under an additive model, suggesting that the odds of being affected by OCD were reduced by 0.74 times with a one-copy increase of the variant C allele. Similarly, the *HTR3B* haplotype with the C allele was associated with OCD subjects in a protective manner. These results imply that the variant C allele of rs1176744 may decrease an individual’s susceptibility to OCD. On the protein level, this functional Tyr129Ser variant results in slow deactivation and desensitization kinetics in variant (p.129S) 5-HT_3_AB receptors compared to in wild-type ones^26^. For receptors composed of both wild-type (p.Y129) and variant (p.129S) 5-HT_3_B subunits, an intermediate maximal response to serotonin has been demonstrated^26^; this finding may correlate with our results showing varying degrees of risk for OCD according to the variant allele dose. Considering that 5-HT_3_B subunits are expressed in the hippocampus and amygdala^27^,^28^,^29^, one possible explanation for our result is that alterations in receptor responsiveness might play a role in fear conditioning and extinction, which could in turn contribute to OCD susceptibility^30^. Moreover, it has been suggested that the AA genotype of this SNP might increase the risk of developing nausea during paroxetine treatment^31^. As nausea is a common side effect of SSRIs, ascertaining those individuals who are likely to develop nausea may facilitate more tailored SSRI treatment strategies. Collectively, the functional variant rs1176744 may underlie the genetic aetiology of OCD and could serve as a predictor of an individual’s drug response.

We also found nominally significant associations between OCD and rs1062613, rs3758987, and rs3782025. Among these, the odds ratio of rs1062613 was 3.51 under a recessive model, which seems to be a large value for a common variant in OCD. Considering that there were only four TT genotypes in controls, our result may have been overfitted^32^. A larger sample of minor allele homozygotes would be needed to obtain a more reliable statistic.

Regarding the onset age of obsessive-compulsive symptoms, we found a significant association between early onset OCD and rs3758987 in male subjects. Although this 5′ upstream variant does not directly affect the amino acid sequence of the encoded protein, this variant may be in LD with a nearby functionally important, but unexplored, polymorphism. On the other hand, this SNP might influence regulatory processes related to *HTR3B* expression. As individuals with early onset OCD may represent a genetically more valid subgroup^25^, further research on the physiological relevance of rs3758987, as well as replication of this association in different populations, is needed.

With regard to the symptom dimensions, the cleaning dimension was significantly associated with two non-synonymous SNPs, rs6766410 and rs6443930, in female subjects. When analysing their putative effects with PolyPhen-2^33^, we found that neither variant was predicted to be damaging. However, in terms of rs6807362 and rs1000952, which were in high LD with rs6766410 and rs6443930, respectively, the analysis revealed that these variants were predicted to be possibly or probably damaging (PolyPhen-2 scores [HumDiv] 0.647 and 0.998, respectively). Thus, the observed association with rs6766410 and rs6443930 may be attributed to other tightly linked functional variants. Notably, rs6766410 was also related to the contamination-based disgust sensitivity. Interestingly, the relationships among rs6766410, the cleaning dimension, and disgust sensitivity were the most robust under an overdominant model in the same direction, in which the heterozygote genotype AC was significantly associated with a reduced risk of the cleaning dimension and with lower contamination-based disgust scores. These results suggest that molecular heterosis may underlie the relationships among this *HTR3C* variant, the cleaning dimension, and disgust sensitivity. According to Comings and MacMurray^34^, our results are likely related to interaction effects between the wild-type and variant 5-HT_3_ receptor subunits.

The gender-specific associations found here are consistent with the findings of previous studies, which revealed sexually dimorphic effects of the genetic variants on OCD^35^,^36^,^37^. Although several of our results did not reach the experiment-wise significance of *α* = 0.0017 (0.05/30 ≈ 0.00167) after Bonferroni correction for three different strata, the *P* values obtained from these stratified analyses were the three most significant ones in the present study. Interestingly, these associations were consistent with the gender differences observed for OCD symptoms, including an earlier onset in men^38^ and more contamination-related symptoms in women^39^. The gender differences in clinical manifestations might thus reflect underlying genetic heterogeneity.

Previous studies have shown that 5-HT_3_ antagonists may be beneficial as OCD treatments^11^,^12^,^13^,^14^,^15^. Hence, the results of this study may have implications for pharmacogenetic studies utilizing 5-HT_3_ antagonists in OCD. It is plausible that genetic variations and the subsequent alterations in receptor function might elicit different responses to 5-HT_3_ antagonists; clarifications regarding the effects of such genetic variations on an individual’s treatment responses may aid in selecting the appropriate treatment options.

To our knowledge, our study on the involvement of *HTR3* in OCD analysed the most individuals. Nonetheless, our study has several limitations. First is the potential for population stratification. As we did not have information on the migration histories of the subjects nor did we include a panel of ancestry-information markers, we could not control the potential effects of an undetected population substructure. Although the considerable degree of genetic homogeneity among the Korean population^40^,^41^ might make bias less likely, the possibility of false-positive associations stemming from population stratification could not be completely excluded. Second, as controls were significantly younger than were individuals with OCD, control subjects may develop obsessive-compulsive symptoms later in life. However, this inevitable factor may have exerted only a trivial effect on the power, because the lifetime prevalence of OCD is ~1–2% and controls had largely passed the mean age of OCD onset^42^. Third, the DS-R scores were obtained from a subset of subjects, which may have reduced the statistical power. Fourth, as the onset age information was collected retrospectively, the potential for recall bias cannot be disregarded. Finally, we investigated only 10 of the *HTR3* polymorphisms, thus associations with other variants may have been missed.

In summary, we found that *HTR3* variants influenced the affected status of individuals with OCD and several of its phenotypes. These findings support that 5-HT_3_ receptors are involved in the pathophysiology and clinical manifestations of OCD. Future studies focusing on the relationships among these *HTR3* variants and the treatment response to 5-HT_3_ antagonists may elucidate whether genetic variations in the 5-HT_3_ receptor also influence the medication response in individuals with OCD.

## Methods

### Subjects

The study sample consisted of 596 individuals with OCD and 599 healthy control subjects. Unrelated individuals with OCD were consecutively recruited from the outpatient department of psychiatry at Severance Hospital, Yonsei University Health System, and diagnosed with the Korean version of the Structured Clinical Interview for Diagnostic and Statistical Manual of Mental Disorders, Fourth Edition (DSM-IV) Axis I disorders^43^ by a trained psychiatrist. Exclusion criteria were as follows: age <19 or >65 years, a lifetime history of psychotic symptoms, history of substance abuse or dependence in the preceding 6 months, or severe organic or neurologic disorders. Subjects with comorbid DSM-IV Axis I disorders were not excluded as long as the obsessive-compulsive symptoms were the main reason for seeking treatment. Gender-matched, unrelated controls were recruited from the local community through advertisements. Controls with a lifetime history of DSM-IV Axis I disorders or neurological disorders were not included in the study. Ethnicity was ascertained through self-reports, and only those subjects who identified themselves as ethnically Korean were enrolled.

The onset age of OCD was defined as the age at which the obsessive-compulsive symptoms first occurred, as recalled by the subject or family members. The threshold for early onset OCD was considered 17 years of age^44^. The severity of the obsessive-compulsive and depressive symptoms was evaluated with the Yale-Brown Obsessive-Compulsive Scale^45^ and Montgomery-Åsberg Depression Rating Scale^46^, respectively. The Yale-Brown Obsessive-Compulsive Scale symptom checklist was employed to identify the following four previously reported symptom dimensions in the meta-analysis^47^: (1) symmetry–symmetry obsessions and repeating, ordering, and counting compulsions; (2) forbidden thoughts–aggressive, sexual, religious, and somatic obsessions and checking compulsions; (3) cleaning–contamination obsessions and cleaning compulsions; and (4) hoarding–hoarding obsessions and compulsions. The presence of a dimension was determined based on a lifetime history of one or more symptoms in the respective category.

This study was approved by the Institutional Review Board of Severance Hospital. The methods were performed in accordance with the approved guidelines. Written informed consent was obtained from each subject at the beginning of the study.

### Disgust scale-revised

Information on individual differences in the sensitivity to disgust was obtained with the Korean version of the DS-R^48^ in subsets of the individuals with OCD (n = 256) and controls (n = 478). The DS-R is comprised of the following three subscales: core disgust scale, animal reminder disgust scale, and contamination-based disgust scale. Core disgust reflects the avoidance or rejection response to disgusting stimuli, including bodily waste products, small animals, and rotting foods. Animal reminder disgust indicates the aversion to stimuli that reminds the individual of the animal origins of humans, including body envelope violations and death. Finally, contamination-based disgust is associated with the perceived risk of disease contagion^6^.

### SNP selection

We selected 10 SNPs from across all of the *HTR3* genes according to either of the following criteria: (1) functional variants annotated in dbSNP (http://www.ncbi.nlm.nih.gov/projects/SNP/) that reside within the regulatory region or alter the amino acid sequence of a protein, or (2) variants previously reported to be related to OCD or other psychiatric disorders. All selected variants had a verified minor allele frequency >0.05 in Asians, as ascertained via the HapMap project database (http://hapmap.ncbi.nlm.nih.gov/; Data Release 28, phase II + III August 10, on NCBI B36 assembly, dbSNP b126).

### Genotyping

Genomic DNA was prepared from blood samples with the QuickGene-mini80 (FUJIFILM, Tokyo, Japan). In a subset of controls (n = 160), DNA was extracted from saliva using the Oragene DNA collection kit (DNA Genotek, Kanata, Ontario, Canada). Genotyping of rs3782025 was performed with the ABI PRISM SNaPShot Multiplex kit (ABI, Foster City, CA, USA) according to the manufacturer’s recommendations. Analyses were conducted using the GeneMapper software (version 4.0; Applied Biosystems, USA). Genotyping of the remaining nine SNPs (rs1062613, rs1176713, rs3758987, rs1176744, rs6766410, rs6807362, rs6443930, rs1000952, and rs7627615) was performed with the TaqMan fluorogenic 5′ nuclease assay (ABI, Foster City, CA, USA) according to the manufacturer’s instructions. Primer sequences and assay IDs are shown in Supplementary Table S2.

### Sample power calculation

Statistical power was evaluated under a dominant genetic model using the Quanto software (version 1.2.4; http://biostats.usc.edu/software); statistical significance was set at *P* < 0.05. Given the available sample size, the statistical power for detecting a risk allele with an effect size of 1.5 ranged from 0.77 to 0.88, depending on the minor allele frequency.

### Statistical analysis

Continuous variables are shown as the mean ± the standard deviation. Group differences in the demographic data were evaluated with Pearson’s χ^2^ tests and independent-samples *t*-tests for categorical variables and continuous variables, respectively. Deviation from Hardy-Weinberg equilibrium was tested using an exact test. The strength of the associations between *HTR3* SNPs and the risk for OCD and its sub-phenotypes (early onset OCD, symmetry, forbidden thoughts, cleaning and hoarding) was examined with binominal logistic regression under dominant, recessive, overdominant, and additive models of inheritance. The analyses were adjusted for age and sex, and the model with the lowest Akaike information criterion was selected as the best-fitting model. The influence of the genetic variants on the disgust sensitivity was evaluated with a one-way multivariate analysis of variance and post-hoc univariate analysis of variance, with the genotype as an independent variable and the DS-R subscales as dependent variables. Analyses were conducted using the R software (version 3.2.1; http://www.r-project.org) and the R package SNPassoc^49^. The overall statistical significance was set at α = 0.005 after Bonferroni correction for the 10 independent SNPs examined. An association was regarded as significant for *P* < 0.005 and nominally significant for 0.005 ≤ *P* < 0.05.

Haploview software (version 4.2; http://www.broad.mit.edu/mpg/haploview) was used to estimate the pairwise LD patterns of the examined SNPs. Haplotype blocks were defined by the solid spine of LD method with a D′ threshold of 0.8^50^. Haplotype-based associations were analysed using the R package haplo.stats^51^, which estimates haplotype frequencies with an expectation-maximization algorithm. Haplotype-specific score statistics were computed to test for associations between the haplotype distributions and OCD under an additive model with the *haplo.score* function. Odds ratios and 95% confidence intervals were calculated using the *haplo.cc* function. A permutation procedure (*n* = 100,000) was performed in order to estimate the corrected significance of the best results.

## Additional Information

**How to cite this article**: Kim, H. W. *et al*. Common variants of *HTR3* genes are associated with obsessive-compulsive disorder and its phenotypic expression. *Sci. Rep.* **6**, 32564; doi: 10.1038/srep32564 (2016).

## Supplementary Material

Supplementary Information

## Figures and Tables

**Table 1:** Characteristics of the *HTR3* variants.

Gene	rs number	Chr	Position^a^	Geno^b^	*P*_HWE_^c^	MAF	Function
*HTR3A*	rs1062613	11	113846006	99.7	0.2134/0.0959/0.7703	0.112	5′ UTR
*HTR3A*	rs1176713	11	113860425	99.0	0.3753/0.0233/0.0247	0.251	Syn
*HTR3B*	rs3758987	11	113775275	98.3	0.8336/0.7298/1.000	0.250	5′ upstream
*HTR3B*	rs1176744	11	113803028	99.5	0.5108/1.000/0.5639	0.228	Non-Syn
*HTR3B*	rs3782025	11	113807607	97.9	0.0327/1.000/0.1261	0.326	Intron 6
*HTR3C*	rs6766410	3	183774762	99.6	0.6104/0.0502/0.0807	0.404	Non-Syn
*HTR3C*	rs6807362	3	183778010	99.3	0.6738/0.4631/0.8816	0.265	Non-Syn
*HTR3D*	rs6443930	3	183754294	99.6	0.9341/0.6772/0.6814	0.444	Non-Syn
*HTR3D*	rs1000952	3	183755822	99.7	0.2992/0.1587/1.000	0.083	Non-Syn
*HTR3E*	rs7627615	3	183818416	99.7	0.8117/1.000/0.9323	0.218	Non-Syn

Chr, chromosome; HWE, Hardy-Weinberg equilibrium; MAF, minor allele frequency; UTR, untranslated region; Syn, synonymous; Non-Syn, non-synonymous.

^a^Information on the chromosomal position is based on NCBI genome build GRCh37.p13. The locations are in reference to NM_000869.5 for *HTR3A*, NM_006028.4 for *HTR3B*, NM_130770.2 for *HTR3C*, NM_182537.2 for *HTR3D,* and NM_182589.2 for *HTR3E*.

^b^Genotyping call rate (%).

^c^*P* value for Hardy-Weinberg equilibrium among controls, individuals with OCD, and the entire sample. Order of *P* values: control subjects/OCD subjects/total subjects.

**Table 2:** Sociodemographic and clinical characteristics of the study sample.

Variable	OCD (*n* = 596)	Controls (*n* = 599)	*P* value
Age, years (range)	29.84 ± 10.52 (19–63)	23.44 ± 3.92 (19–48)	<0.001
Male/Female, *n*	390/206	393/206	0.950
Education, years	13.56 ± 2.38	13.39 ± 1.95	0.169
Onset age of OCD, years	18.48 ± 8.97		
Early onset (≤17years), *n* (%)	328 (55.0)		
Late onset (>17years), *n* (%)	268 (45.0)		
Illness duration, years	11.36 ± 8.30		
Basal Y-BOCS score			
Total	24.92 ± 5.94		
Obsessions	12.67 ± 3.03		
Compulsions	12.24 ± 3.43		
Basal MADRS score	18.59 ± 8.71		
Comorbid diagnosis, *n* (%)			
Affective disorders	77 (12.9)		
MDD (*n* = 46)			
Depressive disorder, NOS (*n* = 21)			
Bipolar I disorder (*n* = 5)			
Bipolar II disorder (*n* = 5)			
Anxiety disorders	35 (5.9)		
Panic disorder (*n* = 22)			
Social phobia (*n* = 9)			
PTSD (*n* = 3)			
GAD (*n* = 1)			
Eating disorders	2 (0.3)		
Tic disorder or Tourette’s disorder	24 (4.0)		
Others	8 (1.3)		
Symptom dimensions, present, *n* (%)			
Symmetry	442 (74.2)		
Forbidden thoughts	506 (84.9)		
Cleaning	437 (73.3)		
Hoarding	201 (33.7)		
DS-R score			
Core disgust	29.55 ± 8.08	28.58 ± 7.18	0.108
Animal reminder disgust	21.63 ± 6.34	18.11 ± 6.52	<0.001
Contamination-based disgust	9.12 ± 4.03	7.07 ± 3.44	<0.001

OCD, obsessive-compulsive disorder; Y-BOCS, Yale-Brown obsessive-compulsive scale; MADRS, Montgomery-Åsberg depression rating scale; MDD, major depressive disorder; Depressive disorder, NOS, depressive disorder, not otherwise specified; PTSD, posttraumatic stress disorder; GAD, generalized anxiety disorder; DS-R, disgust scale-revised.

**Table 3:** Distribution of allelic and genotypic frequencies of *HTR3* SNPs and their associations with the risk of OCD.

SNP	Alleles				Genotypes					
	D/d^a^	OCD^b^	Control^b^	*P* value^c^	OCD^d^	Control^d^	OR_dom_ (95% CI); *P* value	OR_rec_ (95% CI); *P* value	OR_ovd_ (95% CI); *P* value	OR_add_ (95% CI); *P* value
rs1062613	C/T	0.112	0.112	0.9482	474/110/12	467/125/4	1.03 (0.76–1.39); 0.8509	3.51 (1.06–11.63); 0.0296^e^	0.94 (0.69–1.27); 0.6757	1.11 (0.84–1.45); 0.4736
rs1176713	A/G	0.255	0.247	0.6524	340/204/49	339/211/40	1.03 (0.80–1.32); 0.8087	1.11 (0.69–1.79); 0.6564^e^	1.00 (0.77–1.30); 0.9931	1.04 (0.85–1.26); 0.7082
rs3758987	T/C	0.233	0.268	0.0481	345/214/30	315/228/43	0.81 (0.63–1.04); 0.0958	0.57 (0.33–0.97); 0.0365	0.91 (0.71–1.18); 0.4950	0.80 (0.65–0.98); 0.0299^e^
rs1176744	A/C	0.206	0.250	0.0104	376/195/25	337/216/40	0.75 (0.58–0.97); 0.0253	0.46 (0.26–0.82); 0.0068	0.87 (0.67–1.13); 0.2998	0.74 (0.60–0.91); 0.0043^e^
rs3782025	A/G	0.344	0.308	0.0574	253/265/70	290/226/66	1.41 (1.09–1.81); 0.0078^e^	1.21 (0.82–1.79); 0.3276	1.30 (1.01–1.68); 0.0403	1.25 (1.04–1.51); 0.0159
rs6766410	A/C	0.403	0.404	0.9667	200/310/85	208/293/94	1.00 (0.77–1.29); 0.9729	0.80 (0.57–1.14); 0.2161^e^	1.11 (0.87–1.42); 0.3970	0.94 (0.78–1.13); 0.5003
rs6807362	C/G	0.265	0.266	0.9410	318/239/38	321/227/44	1.00 (0.78–1.28); 0.9853	0.79 (0.48–1.30); 0.3579^e^	1.07 (0.83–1.37); 0.6247	0.96 (0.79–1.18); 0.7222
rs6443930	C/G	0.438	0.451	0.5300	185/298/111	179/297/120	1.05 (0.80–1.38); 0.7164	0.93 (0.68–1.26); 0.6249	1.10 (0.86–1.40); 0.4688^e^	1.00 (0.84–1.19); 0.9673
rs1000952	A/G	0.078	0.087	0.4132	509/81/6	494/100/2	0.78 (0.56–1.10); 0.1559	3.19 (0.61–16.62); 0.1410	0.73 (0.51–1.03); 0.0731^e^	0.85 (0.62–1.17); 0.3129
rs7627615	A/G	0.215	0.220	0.7610	366/202/27	364/203/30	0.93 (0.72–1.20); 0.5736	0.83 (0.46–1.51); 0.5478	0.96 (0.74–1.25); 0.7536	0.93 (0.75–1.15); 0.4916^e^

OCD, obsessive-compulsive disorder; SNP, single nucleotide polymorphism; OR, odds ratio; CI, confidence interval; dom, dominant; rec, recessive; ovd, overdominant; add, additive.

^a^Lowercase *d* denotes the less frequent allele.

^b^Minor allele frequencies in individuals with OCD and controls.

^c^*P* values via Pearson’s χ^2^ test for allelic associations.

^d^Number of genotypes in individuals with OCD and controls. Order of genotypes: DD/Dd/dd (d is the minor allele).

^e^Genetic inheritance model with the lowest Akaike information criteria.

**Table 4:** Association between rs3758987 and the onset age.

Genotype	OCD subjects, total				Male subjects				Female subjects			
	Early onset OCD, *n* (%)		OR (95% CI)	*P* value	Early onset OCD, *n* (%)		OR (95% CI)	*P* value	Early onset OCD, *n* (%)		OR (95% CI)	*P* value
	No	Yes			No	Yes			No	Yes		
TT	142 (53.6)	203 (62.7)	1.00		71 (49.3)	156 (65.0)	1.00		71 (58.7)	47 (56.0)	1.00	
TC-CC	123 (46.4)	121 (37.3)	0.66 (0.45–0.97)	0.0347	73 (50.7)	84 (35.0)	0.49 (0.31–0.79)	0.0031	50 (41.3)	37 (44.0)	1.18 (0.61–2.29)	0.6281

OCD, obsessive-compulsive disorder; OR, odds ratio; CI, confidence interval.

**Table 5:** Associations between rs6766410 and rs6443930 and the cleaning dimension.

Genotype	OCD subjects, total				Male subjects				Female subjects			
	Symptoms, *n* (%)		OR (95% CI)	*P* value	Symptoms, *n* (%)		OR (95% CI)	*P* value	Symptoms, *n* (%)		OR (95% CI)	*P* value
	Absent	Present			Absent	Present			Absent	Present		
rs6766410												
AA-CC	67 (42.4)	218 (49.9)	1.00		51 (49.5)	137 (47.9)	1.00		16 (29.1)	81 (53.6)	1.00	
AC	91 (57.6)	219 (50.1)	0.75 (0.52–1.08)	0.1173	52 (50.5)	149 (52.1)	1.08 (0.69–1.70)	0.7389	39 (70.9)	70 (46.4)	0.36 (0.18–0.69)	0.0016
rs6443930												
CC	52 (33.1)	133 (30.4)			42 (40.4)	84 (29.4)			10 (18.9)	49 (32.5)		
CG	68 (43.3)	230 (52.6)			40 (38.5)	146 (51.0)			28 (52.8)	84 (55.6)		
GG	37 (23.6)	74 (16.9)	0.91 (0.70–1.18)	0.4863	22 (21.2)	56 (19.6)	1.21 (0.88–1.67)	0.2361	15 (28.3)	18 (11.9)	0.47 (0.29–0.79)	0.0030

OCD, obsessive-compulsive disorder; OR, odds ratio; CI, confidence interval.

**Table 6:** Estimated haplotype frequencies in individuals with OCD and controls.

Haplotype (*HTR3B*)		Hap-score	OCD %	Controls %	OR (95% CI)	Crude *P* value	Permutated *P* value
rs3758987	rs1176744						
T	A	2.39273	73.6	69.1	1.00 (reference)		
C	C	−2.37972	18.1	22.0	0.77 (0.63–0.95)	0.0173	0.0179
C	A	1.17538	4.9	3.8	1.17 (0.79–1.72)	0.2398	0.2410
T	C	0.28567	2.2	2.0	1.04 (0.59–1.82)	0.7751	0.7699

OCD, obsessive-compulsive disorder; OR, odds ratio; CI, confidence interval.
