# Supplementary material for: Common variants of HTR3 genes are associated with obsessive-compulsive disorder and its phenotypic expression
Source: Sci Rep. 2016 Sep 12;6:32564. doi: 10.1038/srep32564 (PMC5018838; doi:10.1038/srep32564)
Supplement: Supplementary Information [file srep32564-s1.pdf]

Supplementary Information

Common variants of *HTR3* genes are associated with obsessive-compulsive disorder and its phenotypic expression

Hae Won Kim, M.D.[1,2], Jee In Kang, M.D.[1,2], Sang-Hyuk Lee, M.D.[3], Sung Yun Sohn, M.D.[1,2], Eun Hee Hwang, M.S.[2], Su Young Lee, M.D.[4], Se Joo Kim, M.D.[1,2,*]

[1]Department of Psychiatry, Yonsei University College of Medicine, Seoul, Republic of Korea.

[2]Institute of Behavioral Science in Medicine, Yonsei University College of Medicine, Seoul, Republic of Korea

[3]Department of Psychiatry, CHA Bundang Medical Center, CHA University, Seongnam, Republic of Korea

[4]Department of Psychiatry, Cheil General Hospital & Women's Healthcare Center, Dankook University College of Medicine, Seoul, Republic of Korea

*Corresponding author: Se Joo Kim, M.D.

Department of Psychiatry, Yonsei University College of Medicine

50-1 Yonsei-ro, Seodaemun-gu, Seoul 03722, Republic of Korea

Tel.: +82-2-2228-1620; *Fax*: +82-2-313-0891

E-mail: kimsejoo@yuhs.ac

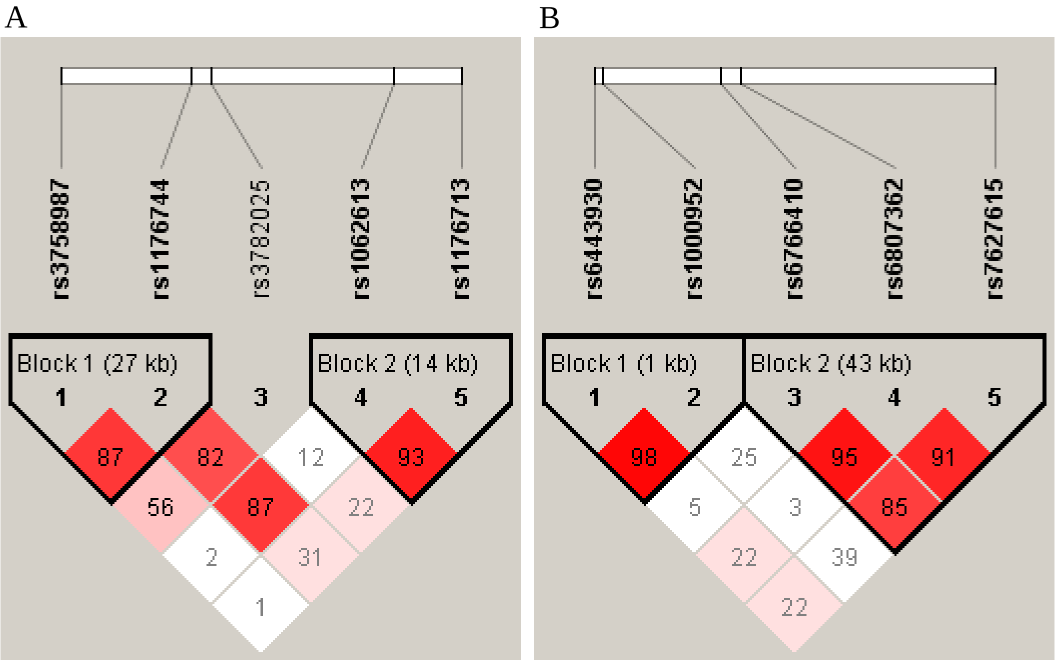

Supplementary Figure S1. Linkage disequilibrium patterns and haplotype blocks estimated with markers that were examined in this study. All numbers in the square represent the pairwise D′ value as a percentile. (A) *HTR3A* (rs1062613, rs1176713) and *HTR3B* (rs3758987, rs1176744, rs3782025). (B) *HTR3C* (rs6766410, rs6807362), *HTR3D* (rs6443930, rs1000952), and *HTR3E* (rs7627615).

Supplementary Table S1. Conditional association results for rs6766410 and rs6443930 and the cleaning dimension

	covariate	rs6766410			rs6443930		
		Total subjects	Male subjects	Female subjects	Total subjects	Male subjects	Female subjects
Model	SNP	OR (95% CI); *P* value			OR (95% CI); *P* value		
Dominant	rs6766410	–	–	–	0.61 (0.40–0.92); 0.0155	0.83 (0.51–1.37); 0.4747	0.31 (0.14–0.69); 0.0021
	rs6443930	1.12 (0.76–1.67); 0.5643	1.62 (1.01–2.60); 0.0460	0.47 (0.21–1.05); 0.0548	–	–	–
Recessive	rs6766410	–	–	–	0.79 (0.47–1.31); 0.3689	0.68 (0.38–1.23); 0.2100	1.33 (0.46–3.86); 0.5863
	rs6443930	0.66 (0.42–1.04); 0.0771	0.95 (0.54–1.68); 0.8674	0.30 (0.13–0.69); 0.0050	–	–	–
Overdominant	rs6766410	–	–	–	0.74 (0.51–1.07); 0.1079	1.07 (0.68–1.68); 0.7856	**0.33 (0.17–0.67); 0.0013**[a]
	rs6443930	1.44 (0.99–2.09); 0.0529	1.61 (1.01–2.56); 0.0420	1.17 (0.61–2.24); 0.6398	–	–	–
Additive	rs6766410	–	–	–	0.73 (0.56–0.97); 0.0297	0.81 (0.58–1.14); 0.2355	0.61 (0.36–1.01); 0.0529
	rs6443930	0.92 (0.71–1.20); 0.5367	1.22 (0.89–1.69); 0.2159	**0.46 (0.27–0.78); 0.0031**[a]	–	–	–

SNP, single nucleotide polymorphism; OR, odds ratio; CI, confidence interval.
[a]Significant *P* values under a genetic model with the lowest Akaike information criteria are highlighted in bold.

Supplementary Table S2. Primer sets and assay IDs used in the experiment

rs number	Primer sequence
rs3782025	Forward: CAGGAAACAGCTATGACCgtggcacatccctgtaaa
	Reverse: TGTAAAACGACGGCCAGTAAAACTGCCCCTACCTACA
	SNP primer: AGGCAAYTACAGGCTAACCTTGG
**rs number**	**Assay ID**
rs1062613	C___7488465_10
rs1176713	C___1372122_10
rs3758987	C__27512451_10
rs1176744	C___7488596_1_
rs6766410	C__26004660_10
rs6807362	C__28948667_10
rs6443930	C__28960641_10
rs1000952	C____109464_10
rs7627615	C__28960643_10

SNP, single nucleotide polymorphism
